# The Gut Microbiota of the Greater Horseshoe Bat Confers Rapidly Corresponding Immune Cells in Mice

**DOI:** 10.3390/ani15050685

**Published:** 2025-02-26

**Authors:** Shan Luo, Xinlei Huang, Siyu Chen, Junyi Li, Hui Wu, Yuhua He, Lei Zhou, Boyu Liu, Jiang Feng

**Affiliations:** 1 College of Animal Science and Technology, Jilin Agricultural Science and Technology University, Jilin 132109, China; 2 College of Life Science, Jilin Agricultural University, Changchun 130117, China; 3 College of Veterinary Medicine, Jilin Agricultural University, Changchun 130117, China; 4 Jilin Provincial Key Laboratory of Animal Resource Conservation and Utilization, Northeast Normal University, Changchun 130024, China

**Keywords:** bats, *Chiroptera*, microbiota, immunomodulation, fecal transplant

## Abstract

Background: Emerging infectious diseases threaten human and animal health, with most pathogens originating from wildlife. Bats are natural hosts for many infectious agents. Previous studies have demonstrated that changes in some specific genes in bats may contribute to resistance to viral infections, but they have mostly overlooked the immune function of the bat gut microbiota. Aims: In this study, we used fecal transplants to transfer the gut microbiota from the Greater Horseshoe Bat (*Rhinolophus ferrumequinum*) into mice treated with antibiotics. The gut microbiota changes in mice were detected using 16S rRNA high-throughput sequencing technology. Flow cytometry was used to detect changes in associated immune cells in the spleen and mesenteric lymph nodes of the mice. Results: The results showed that the gut microbiota of mice showed characteristics of some bat gut microbiota. The Greater Horseshoe Bat’s gut microbiota changed some immune cells’ composition in the spleen and mesenteric lymph nodes of mice and also conferred a faster and higher proportion of natural killer cell activation. Conclusion: This result provides new evidence for the regulatory immune function of bat gut microbiota and contributes to a deeper insight into the unique immune system of bats.

## Simple Summary

Bats are natural hosts for numerous pathogens, and the study of bats that carry pathogens without developing disease helps deepen our understanding of the relationship between immunity and infectious disease. Bat-specific habits and the lack of specific reagents have limited bat immunity-related studies, while existing bat immunity studies have neglected the role of gut microbiota in modulating immunity. In this study, we first treated specific pathogen-free (SPF) C57BL/6 mice with a complex antibiotic solution for 7 consecutive days to obtain pseudo-sterile mice and then transplanted the gut microbiota of bats into the mice through fecal microbiota transplantation (FMT), thereby bypassing the dilemma of studying the gut microbiota of wildlife. The results showed that on days 7 and 14 after FMT, the gut microbiota of the Greater Horseshoe bat, a widely distributed insectivorous bat, could regulate immune cells in mice, which exhibited a rapid innate immune response. This result improves our understanding of the unique immune system of bats and emphasizes the importance of bat gut microbiota in immunity.

## 1. Introduction

There are many kinds of wild animals, and bats have attracted special attention due to their association with many fatal infectious diseases [1]. Bats are nocturnal, resting during the day in environments such as tree canopies, caves, rock crevices, houses, and man-made canals, and flying out in the evening to hunt for food. The diet of bats is complex because of the large number of different species and mainly divides into several categories, such as insect-eating, fruit-eating, nectar-eating, meat-eating, blood-eating, and omnivorous [2]. Since the outbreak of severe acute respiratory syndrome (SARS) in 2002 and severe acute respiratory syndrome coronavirus 2 (SARS-CoV-2) in 2019, researchers have paid more attention to the study of bats that carry pathogens without disease [3,4]. However, due to bats’ strong flight ability, nocturnal mobility, and the lack of corresponding specific antibodies and other reagents, research progress on bat immunity is limited.

The stability of the immune system is affected by many factors, such as genetic predisposition, nutrition, environment, and gut microbiota. Intestinal homeostasis is crucial for the normal life activities of the body. The long-term co-evolution between the immune system and gut microbiota maintains intestinal homeostasis [5]. In addition, gut microbiota plays a key role in developing the host immune system and regulating immune homeostasis [6,7]. The development of the immune system depends not only on the development of secondary immune organs, such as lymph nodes and Peyer’s patches, but also on immune cells that normally recognize antigens after priming. Gut microbiota and its metabolites play an important role in this process [8]. Studies in germ-free mice have shown that the immune system is incompletely developed due to the lack of gut microbiota. This is manifested by the reduced size of the thymus, alterations in the structure of the spleen, the reduction of the size and number of cells in the internal splenic germinal centers, the low secretion of secretory immunoglobulin A (SIgA), a decrease in immune system function, a decrease in the number of Peyer’s patches and their germinal centers, and so on [9,10].

There are a number of hypotheses as to why bats carry pathogens without clinical signs. Some suggest that this is due to their high body temperatures associated with flight, which restrict pathogen reproduction, while others believe that this may be attributed to a balance of enhanced host defense and immune tolerance in bats, and others hypothesize that bats infected with the virus quickly mount an immune response to limit replication of the virus and, therefore, do not show clinical symptoms [11,12,13]. The existing research on bat immunology mostly focuses on bat autoimmune genes and immune cells. Current studies have found that bats have lymphoid organs and tissues similar to those of other mammals, such as the thymus, bone marrow, spleen, and lymph nodes [14,15]. Studies on *Pteropus alecto* (*P. alecto*) showed that the proportion of CD4^+^ and CD8^+^T lymphocytes varied in different parts of the bat’s body. In the spleen, CD8^+^T lymphocytes were dominant, while in the blood, the opposite was true [16]. In the exploration of bat-specific immunity, gut microbiota is an important link that cannot be ignored [17]. Unlike other mammals, the microbiome of the bat gut appears to be composed primarily of Proteobacteria, followed by Firmicutes and Bacteroidetes [18]. However, unlike other bats that have been reported, the great evening bat (*Ia io*) had a higher abundance of Firmicutes than Proteobacteria, suggesting that there may be significant differences in gut microbiota between bats [19]. Like other mammals, the gut microbiota of bats is affected by many factors, such as genetics, food habits, and seasonal and geographical changes. For example, it was found that the α diversity of the gut microbiota of *Rhinolophus ferrumequinum* (*R. ferrumequinum*) changed with the seasons, and the order from high to low was late summer, early spring, early winter, and early summer [20]. The periodic changes in the gut microbiota composition of *R. ferrumequinum* may be caused by fasting during hibernation, while the unique gut microbiota of *Ia io* may be caused by changes in feeding habits (the conversion between insectivores and birds).

Despite a series of advances in the study of the bat gut microbiota, most of the existing research has focused on the structural aspects, and little is known about the functions of the bat gut microbiota, especially in the regulation of the immune system. To the best of our knowledge, only a few experimental studies on gut microbiota–immunity interrelationships have been conducted in bats, but only limited detection was possible due to specific reagents, e.g., LPS causes an inflammatory response and contributes to changes in the gut microbiota of fruit bats, and vampire bats exhibit a high abundance of *Edwardsiella* sp. in the gut microbiota after a shift of food source (with correlation to IgG) [21,22]. Our previous study provided the first experimental evidence that the gut microbiota of bats can regulate some immune cells in mice [23]. However, considering the large number of species (more than 1400 species), wide distribution, and complex feeding habits of bats, the functions of their gut microbiota may be inconsistent, and the conclusions drawn from studies on a single species are often too narrow. Therefore, in this study, we investigated the effect of the gut microbiota of another smaller insectivorous bat, *R. ferrumequinum*, on the composition of immune cells in the spleen and mesenteric lymph nodes of mice. We transplanted the gut microbiota of *R. ferrumequinum* into antibiotic-treated mice using fecal microbiota transplantation (FMT). The characteristics of the gut microbiota were detected by 16S rRNA high-throughput sequencing. Ratios of T cells (CD3, CD4, and CD8), B cells (CD45R), and natural killer cells (NK1.1 and NK1.1CD45R) in the spleen and mesenteric lymph nodes (MLN) were measured using flow cytometry to confirm the immunoregulatory effect of the gut microbiota of *R. ferrumequinum*.

## 2. Materials and Methods

### 2.1. Study Design, Mice, Collection, and Preservation of Feces

**Figure 1:**
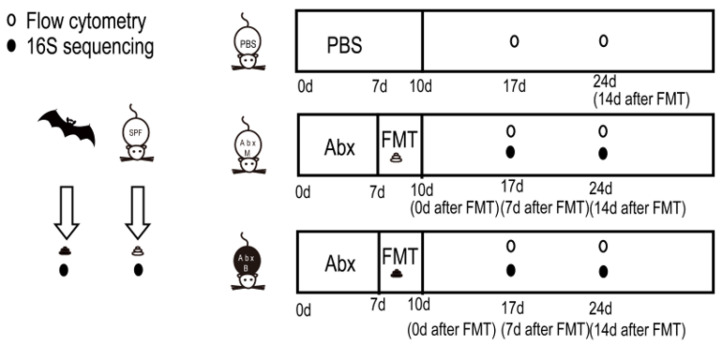
Study design sketch.

The experimental design is shown in Figure 1. In short, the gut microbiota of mice was cleared using compound antibiotics, and then the intestinal tract was emptied with a compound PEG solution to prepare pseudo-sterile mice. The feces of the mice (AbxM group) and the feces of *R. ferrumequinum* (AbxR group) were transplanted into the mice. The microbial structure of the feces of the mice after transplantation was analyzed using 16S rRNA high-throughput sequencing, and the status of related immune cells in the mice was detected using flow cytometry on the 7th day (AbxM7 group and AbxR7 group) and the 14th day (AbxM14 group and AbxR14 group). 

The 3-week-old female C57BL/6 SPF mice were provided by Beijing Huafukang Biotechnology Co., Ltd. (Beijing, China). The mice were fed in the animal room, given food sterilized using cobalt 60 radiation and double-distilled water, and fed freely, with the ambient temperature maintained at 22 ± 2 °C and the light cycle alternating every 12 h.

The fresh feces of *R. ferrumequinum* were collected in Ji’an City, Jilin Province, China, in July 2023. The sampling method is as described previously [23]. In short, bats are collected in kraft paper bags that have been sterilized by high-pressure steam. Fresh feces in the bags are collected every 5 min for 30 min. The fresh feces are placed in PBS buffer (Hyclone, Logan, UT, USA) with 20% glycerol (Hyclone, Logan, UT, USA), frozen in liquid nitrogen as soon as possible, and transferred to a −80 °C refrigerator for frozen storage. Fresh feces from SPF mice are collected from the same batch of SPF mice.

### 2.2. Fecal Microbiota Transplantation

The method of FMT was as described previously. In short, mice were administered compound antibiotics (Ampicillin sodium salt 200 mg/kg, neomycin sulfate 200 mg/kg, metronidazole 200 mg/kg, and Vancomycin hydrochloride 100 mg/kg) (Sigma-Aldrich, St. Louis, MO, USA) for 7 days, followed by 1.5 mL of compound Polyethylene Glycol (PEG) (Sigma-Aldrich, St. Louis, MO, USA) solution administered 5 times within 2 h on the 8th day. Oral administration was used to clear the intestinal residue, and then fecal bacteria transplantation was carried out for three consecutive days, and the transplanted bacterial solution was administered. The operation of preparing the fecal bacteria transplantation solution was completed in an anaerobic operation room with CO_2_ and N_2_ flowing through. The frozen feces were quickly thawed in a 37 °C water bath and mixed. After three washes with PBS containing 20% glycerol PBS and centrifugation (7000× *g*), the concentration was adjusted to 50 mg/mL and then frozen in the refrigerator at −80 °C until use [23,24].

The fecal bacterial composition reflects changes in gut microbiota after transplantation. Thirty mice were equally and randomly divided into three groups: PBS (*n* = 10), AbxM (*n* = 10), and AbxR (*n* = 10). On days 7 and 14 after FMT, feces were taken for subsequent testing and labeled as AbxM7 (*n* = 5), AbxR7 (*n* = 5), AbxM14 (*n* = 5), and AbxR14 (*n* = 5). Fecal samples from each group were flash-frozen in liquid nitrogen immediately after collection and then cryopreserved at −80 °C.

### 2.3. 16S rRNA Gene Sequencing

Extraction of microbial community genomic DNA from fecal samples was performed using the E.Z.N.A. ^®^Soil DNA kit (Omega Bio-tek, Norcross, GA, USA) according to the manufacturer’s instructions. The hypervariable region V3-V4 of the bacterial 16S rRNA gene was amplified using primer pairs 338F(5′-ACTCCTACGGGAGGCAGCAG-3′) and 806R(5′-GGACTACHVGGGTWTCTAAT-3′) [25]. PCR reactions were performed following the protocol described previously [19]. The PCR products were extracted and purified from a 2% agarose gel using an AxyPrep DNA Gel Extraction kit (Axygen Biosciences, Union City, CA, USA), and the Quantus ™ Fluorescence instrument (Promega, Madison, WI, USA) was used for quantitative analysis. Purified amplicons were pooled in equimolar amounts and paired-end sequenced on an Illumina MiSeq PE300 platform (Illumina, San Diego, CA, USA) according to the standard protocols provided by Majorbio Technology Co., Ltd. (Shanghai, China).

The processing and analysis of raw data were performed using the cloud platform (www.majorbio.com. accessed on 1 January 2025) of Shanghai Meiji Biomedical Technology Co. Briefly (Shanghai, China), FLASH (v1.2.11) was used for merging; quality filtering, denoising, and merging were performed using DADA2, which is a plugin for QIIME2 (version 2020.2); classification was performed using q2-feature-classier64 and the Silva database; alpha diversity analysis was performed using Q2 diversity; similarity between microbial communities was determined using nonmetric multidimensional scaling (NMDS); the linear discriminant analysis (LDA) effect size (LEfSe) was analyzed via LDA set at 3.5; and, finally, functional prediction was completed using PICURSt2 [26,27,28,29,30].

### 2.4. Flow Cytometry

On the 7th and 14th days after FMT, 5 mice in each group were randomly selected to prepare spleen and MLN single-cell suspensions, as previously described [31]. The cell suspension was sealed with FC block antibody, and then cell death dye, CD3e, CD4, CD8α, CD45R, and NK1.1 antibody were washed twice for detection. All antibodies used were purchased from BD Bioscience. Samples were assayed using BD LSR Fortessa (BD Biosciences, San Jose, CA, USA), and the data were analyzed with Flowjo10 (Treestar, Ashland, OR, USA).

### 2.5. Statistical Analyses

Statistical analyses were performed using GraphPad Prism 8.0 software (GraphPad Software, San Diego, CA, USA). For flow cytometry data, we first tested whether the data conformed to a normal distribution using the Shapiro–Wilk test. Next, one-way ANOVA, followed by Tukey’s test, was used when the data conformed to a normal distribution, and the Kruskal–Wallis test, followed by Tukey–Kramer, was used if they did not conform to a normal distribution. All tests were two-sided. Alpha diversity analysis of microbiome data was performed using the Kruskal–Wallis test, followed by Dunn’s test.

## 3. Results

### 3.1. Microbial Changes in Feces of Mice After FMT

**Figure 2:**
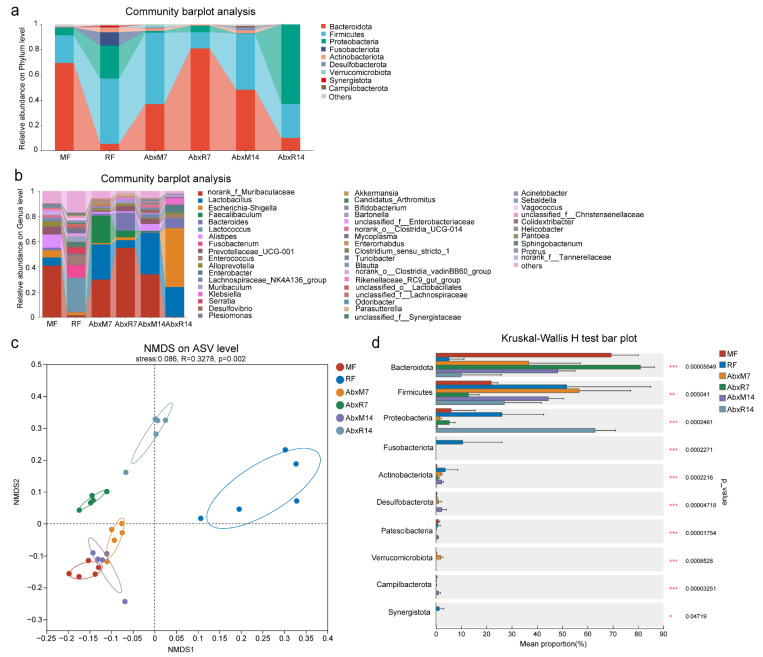
Changes in the distribution of gut microbiota in mice. Community bar plot analysis of phylum (**a**) and genus (**b**). (**c**) NMDS based on unweighted UniFrac distance matrices. (**d**) Relative abundance distributions of the phylum. MF: Feces from mouse donors; RF: Feces from bat donors; AbxM7: Feces on day 7 in mice receiving fecal transplants from mouse donors; AbxR7: Feces on day 7 in mice receiving fecal transplants from bat donors; AbxM14: Feces on day 14 in mice receiving fecal transplants from mouse donors; AbxR14: Feces on day 14 in mice receiving fecal transplants from bat donors.*, *p* < 0.05; **, *p* < 0.01; ***, *p* < 0.001.

Throughout the 14-day observation period following fecal microbiota transplantation, no overt clinical symptoms were observed in any of the experimental groups. The Chao and Shannon indices of the gut microbiota in bats were significantly lower than those in mice, indicating that the richness and diversity of the gut microbiota in bats were lower than those in mice (Chao index, *p* < 0.001, Shannon index, *p* < 0.01, Supplementary Figure S1a,b). The results of the bar chart showed that there were significant differences in the gut microbiota between bats and mice. In addition, the composition of gut microbiota in mice after FMT changed significantly on the 7th and 14th days. On the 14th day, it was similar to bat donors, showing higher levels of *Proteobacteria* and *Firmicutes*. In addition, the abundance of *Enterobacteriaceae* was higher in the bat donor and AbxR groups, while the abundance of *Muribaculaceae*, *Prevotellaceae*, *Lachnospiraceae_NK4A136_group*, *Muribaculum*, *Clostridia_UCG-014*, *Enterorhabdus*, and *Odoribacter* was lower (Figure 2a,b). NMDS results showed that FMT changed the composition of the intestinal microbiome in mice, with the AbxR7 and AbxR14 groups moving away from the MF group and toward the RF group on the matrix (Figure 2c). Multiple group comparative analysis using the Kruskal–Wallis rank sum test showed that compared with the 7th day after transplantation, the fecal microorganisms on the 14th day after transplantation were more similar to those of bat donors, showing lower *Bacteroidota* (*p* < 0.001, Figure 2d) levels and a higher abundance of *Firmicutes* (*p* < 0.01, Figure 2d) and *Proteobacteria* (*p* < 0.001, Figure 2d). Comparative analysis of multiple groups showed that compared with the 7th day after transplantation, the fecal microorganisms on the 14th day after transplantation were more similar to those of bat donors, showing lower levels of Bacteroidota (*p* < 0.001, Figure 2d) and a higher abundance of *Firmicutes* (*p* < 0.01, Figure 2d) and Proteobacteria (*p* < 0.001 for both, Figure 2d).

**Figure 3:**
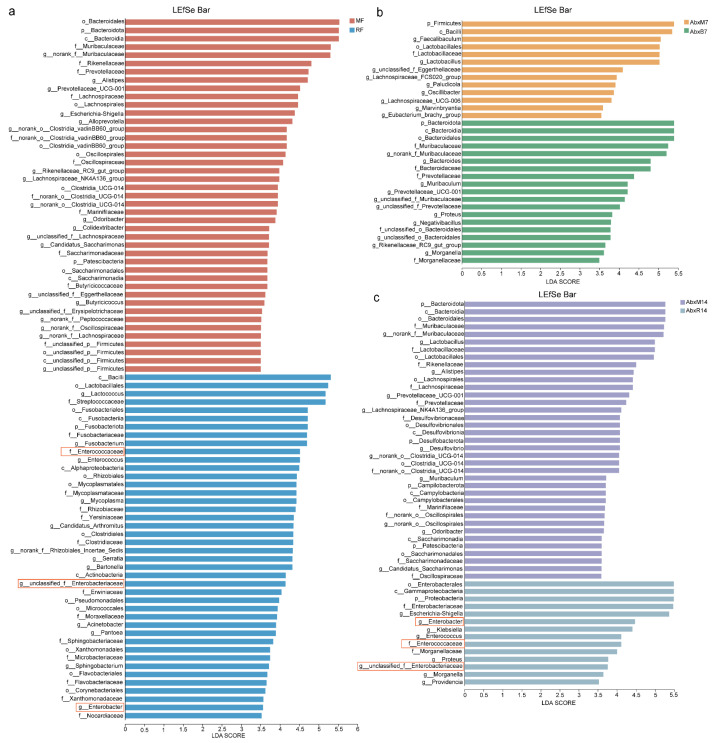
Changes in LEfSe analysis of gut microbiota after FMT. (**a**) LEfSe bar graphs of microorganisms from the MF and RF groups. (**b**) LEfSe bar graphs of microorganisms from the AbxM7 and AbxR7 groups. (**c**) LEfSe bar graphs of microorganisms from the AbxM14 and AbxR14 groups. Box: Representative flora with LDA over 3.5 in RF and AbxR14 groups. MF: Feces from mouse donors; RF: Feces from bat donors; AbxM7: Feces on day 7 in mice receiving fecal transplants from mouse donors; AbxR7: Feces on day 7 in mice receiving fecal transplants from bat donors; AbxM14: Feces on day 14 in mice receiving fecal transplants from mouse donors; AbxR14: Feces on day 14 in mice receiving fecal transplants from bat donors.

**Figure 4:**
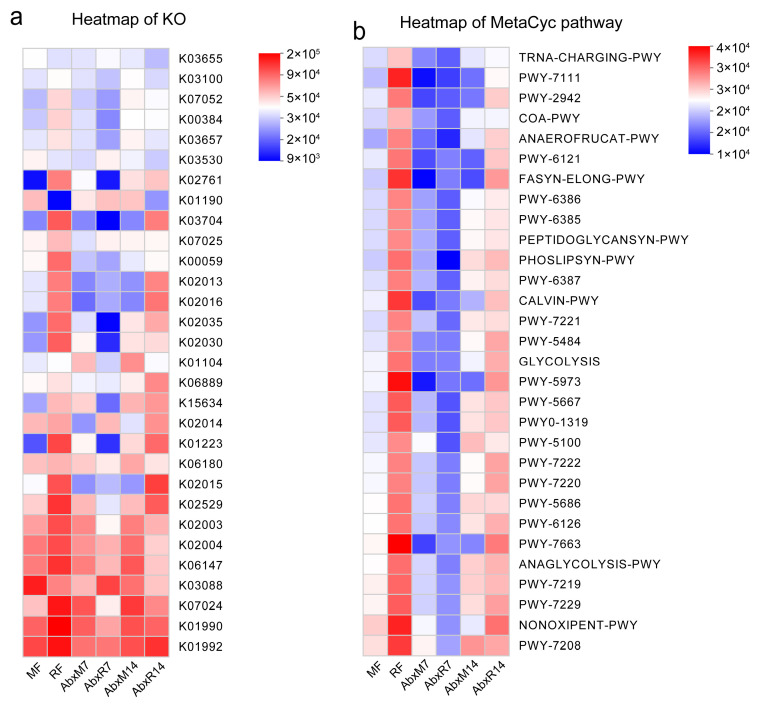
PICRUSt2 analysis of gut microbiota after FMT. (**a**) KO heatmap for PICRUSt2 analysis. (**b**) MetaCyc pathway heatmap for PICRUSt2 analysis. MF: Feces from mouse donors; RF: Feces from bat donors; AbxM7: Feces on day 7 in mice receiving fecal transplants from mouse donors; AbxR7: Feces on day 7 in mice receiving fecal transplants from bat donors; AbxM14: Feces on day 14 in mice receiving fecal transplants from mouse donors; AbxR14: Feces on day 14 in mice receiving fecal transplants from bat donors.

The results of the LEfSe analyses showed an enrichment of different bacteria between the groups. *Bacilli*, *Lactococcus*, *Streptococcaceae*, *Enterococcaceae*, *Nocardiaceae*, *Enterobacter*, *Enterobacteriaceae*, and *Alphaproteobacteria* were found in bat donors compared to mouse donors, and *Enterococcus*, *Enterococcaceae*, etc. had higher LDA values (Figure 3a). In addition, there was an enrichment of different bacteria across all groups. Higher LDA values in AbxR7 compared to AbxM7 were observed for *Bacteroidota*, *Bacteroidia*, *Muribaculaceae*, *Bacteroides*, *Morganella*, *Proteus*, *Muribaculum*, and *Prevotellaceae* (Figure 3b). After 14 days of fecal transplantation, the AbxR group of *Enterobacterales*, *Gammaproteobacteria*, *Proteobacteria*, *Enterobacteriaceae*, *Escherichia-Shigella*, *Enterobacter*, *Klebsiella*, *Enterococcus*, *Enterococcaceae*, *Morganellaceae*, *Proteus*, *Morganella,* and *Providencia* had higher LDA values (Figure 3c). Although evolutionary analysis plots of differential flora showed some crossover and overlap between groups, overall, the results for bat fecal donors and their acceptors differed from the structure of mouse cecal donors and acceptors (Supplementary Figure S1c). Predictive analysis of KEGG function based on PICRUSt2 showed functional differences between the bat and mouse gut microbiota, with the bat gut microbiota being more enriched for K02015, K01223, K15634, K02030, K02035, K02016, K02013, K00059, K07025, K03704, K02761, K00384, and K07052 (Figure 4a). The mouse gut microbiota was enriched in the K01190 pathway. In addition, the AbxR14 group exhibited more similar functions to those of the bat donors, such as being equally enriched in K02015, K01223, K15634, K02030, K02035, K02016, K02013, K03704, and K02761 (Figure 4a). In contrast, MetaCyc’s functional prediction analysis showed that the bat donor was functionally similar to AbxR14, enriched for PWY-7208, NONOXIPENT-PWY, PWY-7229, PWY-7219, ANAGLYCOLYSIS-PWY, PWY-7663, PWY-6126, PWY-7220, PWY-7222, PWY0-1319, PWY-5667, PWY-5973, GLYCOLYSIS, PWY-5484, CALVIN-PWY, PHOSLIPSYN-PWY, FASYN-ELONG-PWY, and others (Figure 4b).

### 3.2. Immunomodulatory Effect of Bat Gut Microbiota on Mice

Given the complex interactions between gut microbiota and immune cells, we performed flow cytometry to detect changes in the proportions of T cells (CD3^+^ CD4^+^ CD8^+^), B cells (CD45R^+^/B220^+^), and natural killer cells (NK1.1^+^ and activated natural killer cells NK1.1^+^CD45R^+^) within the spleens and MLN of mice at 7 and 14 days after FMT. We have provided the circle-gate strategy for flow cytometry in the Supplementary Materials (Supplementary Figure S2).

**Figure 5:**
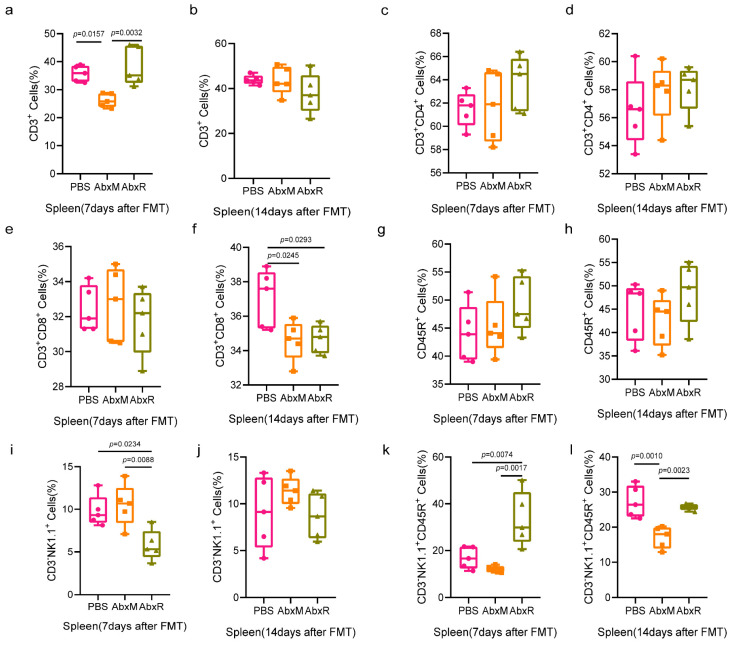
Changes in adaptive immunity (T and B cells) and natural killer cells in the spleen. Control: PBS group; AbxM: a group receiving mouse fecal transplants; AbxR: a group receiving bat fecal transplants. (**a**–**h**) Changes in the proportion of lymphocytes in the spleen after FMT. (**i**–**l**) Changes in the proportion of NK cells in the spleen after FMT. Five mice per group, with each point representing a sample from one mouse. A significance level of *p* < 0.05 was considered significantly different from each group. Data were tested for statistical significance using one-way ANOVA and a subsequent Tukey test. PBS: the control group without FMT and with equal amounts of PBS instilled throughout; AbxM: the group of mice receiving fecal transplants from mouse donors; AbxR: the group of mice receiving fecal transplants from bat donors.

Results in the spleen showed that the bat gut microbiota recovered the proportion of CD3^+^ cells reduced by antibiotic treatment by day 7, whereas the mouse gut microbiota did not, and this recovery disappeared by day 14 (Figure 5a,b). CD3^+^CD4^+^ T cells and CD45R^+^ B cells did not show significant changes throughout the experimental period (Figure 5c,d,g,h). CD3^+^CD8^+^ T cells showed a decrease at day 14 (Figure 5e,f). For natural killer cells, the AbxR group showed a decrease on day 7 after fecal transplantation, which disappeared by day 14 (Figure 5i,j). Interestingly, the performance of activated natural killer cells was different from that of natural killer cells, with a higher percentage of activated natural killer cells in the AbxR group than in the other two groups at day 7, and essentially the same in the AbxR and PBS groups at day 14, both higher than in the AbxM group (Figure 5k,l).

**Figure 6:**
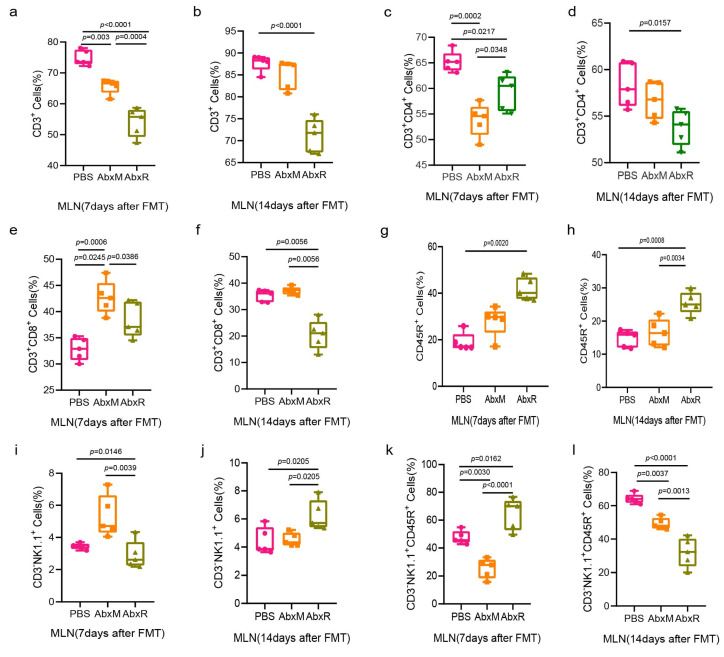
Changes in adaptive immunity (T and B cells) and natural killer cells in the MLN. (**a**–**h**) Changes in the proportion of lymphocytes in the MLN after FMT. (**i**–**l**) Changes in the proportion of NK cells in the MLN after FMT. The experimental groups were the same as the spleen (see Figure 3). Five mice per group, with each point representing a sample from one mouse. A significance level of *p* < 0.05 was considered significantly different from each group. Data (**a**–**f**,**i**–**l**) were tested for statistical significance using one-way ANOVA, which was followed by Tukey’s test, and (**g**,**h**) were tested using the Kruskal–Wallis test and, subsequently, Dunn’s test. PBS: the control group without FMT and with equal amounts of PBS instilled throughout; AbxM: the group of mice receiving fecal transplants from mouse donors; AbxR: the group of mice receiving fecal transplants from bat donors.

In mesenteric lymph nodes, both the AbxM and AbxR groups showed a decrease in the levels of CD3^+^ and CD3^+^CD4^+^ T cells, which recovered in the AbxM group on day 14 (Figure 6a–d). On day 7 after FMT, the AbxM group showed a significant increase in CD3^+^CD8^+^ T cells, which returned to normal on day 14, while the AbxR group showed a nonsignificant increase on day 7 and a significant decrease on day 14 (Figure 6e,f).

Significant elevation of CD45R^+^ B cells was observed in the AbxR group on both day 7 and day 14 (Figure 6g,h). Natural killer cells in the AbxM group showed a significant increase on day 7 and returned to normal on day 14, while in the AbxR group, they were normal on day 7 but increased on day 14 (Figure 6i,j). For activated natural killer cells, the AbxM group was significantly lower than the PBS and AbxR groups on day 7, and lower than the PBS group but higher than the AbxR group on day 14. The activated natural killer cells in the AbxR group were higher than those in the other two groups on day 7 and lower than those in the other two groups on day 14 (Figure 6k,l).

## 4. Discussion

The mouse is a commonly used animal model that is cheap and stable. It has been widely used in research as an alternative to humans and other animals and provides a good alternative for studying gut microbiota across many species. Studies have been conducted to successfully transplant gut microbiota from wildlife to mice via FMT [32,33,34]. Therefore, we believe that mice are suitable for studies related to the transplantation of bat gut microbiota and can serve as an animal model within the constraints of current technological conditions.

The gut microbiota of bats differs from that of other mammals, with low alpha diversity and high levels of *Proteobacteria* or *Firmicutes*. Some researchers believe that this is an adaptation brought about by flight because it has similarities with the structure of bird gut microbiota [35,36]. Our study also confirmed these results. In addition, the results of the LEfSe analysis showed that the AbxR group of mice successfully inherited *Morganellaceae*, *Enterobacteriaceae*, *Enterobacter*, and *Escherichia-Shigella*, all of which belong to the phylum Proteobacteria, from bat donors. The relative abundance of Proteobacteria in the feces of mice with gut microbiota from *R. ferrumequinum* was higher, indicating that the mice successfully exhibited bat gut microbiota characteristics and achieved successful microbiota transplantation. Proteobacteria is generally considered a phylum of bacteria with multiple functions, accounting for a relatively high proportion of flying organisms, such as birds and bats [36]. However, the relative abundance of Fusobacteriota in bats is relatively high, and no Fusobacteriota was found in mice after FMT (AbxR group), which may be due to differences in genetic background and diet between bats and mice. Therefore, mice transplanted with bat feces do not retain the complete bat gut microbiome and can only exhibit some of its characteristics, such as lower alpha diversity, and in NMDS analysis, the coordinate position is closer to the bat donor rather than overlapping, which is also in line with the significant biological background differences between mice and bats. In addition, given the limitations of PICRUSt2, it is prudent to assume that the function of the gut microbiota of *R. ferrumequinum* may differ from that of the mouse gut microbiota and that the AbxR group on day 14 after FMT was closer to the bat in some of its functions.

Studies of bat immunity commonly agree that bats do not exhibit significant clinical symptoms when infected with viruses or other microorganisms, especially certain pathogens that have a substantial impact on humans and other animals [37,38,39]. Experimental challenges of individual bats and studies of bat cell line infections support this view, which indicates that bats may tolerate viral infections even better than other mammals [40]. Mammals have extensive, diverse, and highly active gut microbiota that co-evolve with the host through continuous interrelationships and play a role in behavior, nutrition, and immunity [5]. However, it is difficult to directly identify and validate the function of wildlife gut microbiota, which may result from the harsh environmental conditions in which wildlife live, the complexity of their biological backgrounds, the lack of targeted reagents, and the fact that many are wild species in need of protection. In this study, our results demonstrate that the gut microbiota from the Greater Horseshoe Bat has an immunomodulatory function in mice, as evidenced by alterations in T and B cells, as well as activation of natural killer cells. The maintenance of host immune homeostasis and resistance to infection is known to be dependent on the interaction between the gut microbiota and the host immune system [41]. In the normal local environment of the intestine, the innate immune cells and the gut microbiota cooperate with each other to accomplish intestinal homeostasis. Goblet cells secrete mucus, defensins, and other substances to isolate the intestinal epithelial cells from the gut microbiota, preventing the intestinal epithelial cells from directly contacting certain conditionally pathogenic bacteria in the gut microbiota that may cause damage. Antibiotics cause dramatic changes in the gut microbiota, resulting in the translocation of gut microbiota and dysregulation of immune cells, especially those key to adaptive immunity, such as natural killer cells, CD3^+^CD4^+^ T cells, and CD3^+^CD8^+^ T cells. Thus, microorganisms are essential for the reconstitution of immune cells, which are vital for the body’s resistance to disease, injury, and tissue reconstruction [42,43,44]. Our results show that the transplantation of gut microbiota could partially restore immune cells that are dysregulated in the presence of antibiotics, which is in accordance with previous studies [45]. In addition, the results of this study indicate that the gut microbiota of *R. ferrumequinum* exhibits rapid immune cell modulation, which is similar to that observed in our previous study of the gut microbiota of *Ia io* but differs from the greater hoofed bat gut microbiota in targeting certain immune cells.

The spleen is the largest secondary immune organ in the body, and the gut microbiota of the horsetail jughead bats rapidly restored the proportion of CD3^+^ T cells in the spleen, showing a faster immune-regulating ability than that of the mouse gut microbiota, which is the same as that of *Hipposideros armiger* [46]. In a study on *P. alecto*, it was found that the percentage of T cells in the spleen was higher than that of B cells, with a higher percentage of T cells and a lower percentage of B cells in the spleen of *P. alecto* compared to mice [47]. The results of the present study similarly showed a higher percentage of T cells in the spleen; however, the need for this trend to disappear primarily at post-FMT time 14 suggests that the bat gut microbiota may have contributed to the higher percentage of T cells in the spleen, but it is possible that this functional flora did not successfully colonize the mouse gut to continue its role. The results for NK cells in the spleen showed a lower percentage of natural killer cells on day 7, which recovered on day 14, while the percentage of activated natural killer cells was higher on day 7. This is in contrast to the immunomodulatory role of the gut microbiota of *Hipposideros armiger* and may be due to differences in the structure of the gut microbiota among different species of bats and differences in their function. In general, gut microbiota allows for immunomodulation in a number of ways. It has been found that *Lachnospiraceae* produces butyrate, which inhibits interferon (IFN)-γ secretion of CD8^+^ T cells by suppressing stimulators of IFN gene (STING) activation in dendritic cells (DCs). It is important to note that it has also been found that butyrate produced by gut microbiota also enhances the activity of CD8^+^ T cells by upregulating IFN-γ [48]. This seemingly contradictory result is also seen in other immune cells, such as B cells and NK cells, which illustrates the complexity of the immune regulatory function of gut microbiota. In addition, in this study, it is not clear which components of the gut microbiota lead to changes in immune cells, necessitating further in-depth exploration in the future.

MLN are an important part of the gut-associated lymphoid tissue (GALT) and play an important role in intestinal immunity. Natural killer cells are important innate immune cells in the body, with the function of recognizing and clearing tumor cells and virus-infected cells, as well as secreting factors, such as IFN-γ, granulocyte-macrophage colony-stimulating factor and various chemokines [49,50]. It is important to note that natural killer cells may be suppressed in bats, as hypothesized based on genomic studies of natural killer cell receptors in *Rousettus aegyptiacus* [51]. The results of the study of natural killer cells in the MLN showed that the gut microbiota restored the percentage of natural killer cells and activated natural killer cells more rapidly in the early stages, whereas an elevated percentage of natural killer cells and a reduced percentage of activated natural killer cells occurred in the later stages. This suggests that the gut microbiota may be involved in the immune homeostasis of the host, in conjunction with the bats’ own genetic traits, allowing the bats to maintain a clinically asymptomatic state, and this rapidly regulated immune response supports the hypothesis that bats have balanced enhanced host defenses and immune tolerance. Flow cytometry results in the AbxR group showed decreased CD3^+^CD8^+^ T cells (the decrease appeared on day 14) and activated natural killer cells (with a decrease on day 7 and recovery on day 14), as well as earlier natural killer cell activation. We suggest that activated natural killer cells might secrete more cytokines, such as iIFN-γ, which is consistent with studies indicating that bats maintained higher than baseline levels of interferon [52,53,54]. This result indicates that the gut microbiota of *R. ferrumequinum* may rapidly activate cells associated with innate immunity at an early stage, which is consistent with the rapid response of innate immunity demonstrated in studies on viral tolerance in bats and suggests that the bat gut microbiota may play an important role in innate immunity. Additionally, we noted a decrease in the ratio of CD3^+^CD8^+^ T cells, which we hypothesized could be a result of the regulation of bat gut flora. This is because, in general, bacterial colonization does not lead to the depletion of CD3^+^CD4^+^ T cells or CD3^+^CD8^+^ T cells, especially when no clinical signs are observed. Due to the lack of reports on immune cells in bat GALT, we are unable to make comparisons with the bats themselves; however, we note that recent studies are producing monoclonal antibodies for bats for use in flow cytometry, which may accelerate the understanding of immunity in bats [55].

The ability of the bat gut microbiota to rapidly modulate immune cells and initiate in vivo immunity supports the hypothesis that bats have rapid and robust in vivo immunity to limit pathogens, as well as the hypothesis that bats have balanced enhanced host defenses and immune tolerance. The gut microbiota of *R. ferrumequinum* maintained lower than normal levels of CD3^+^ T cells and CD3^+^CD4^+^ T cells, along with elevated CD45R^+^ cells and natural killer cells in the MLN. This result is also similar to that of a previous study of the greater hoofed bat, which showed that, like insectivorous bats, the gut microbiota of *Hipposideros armiger* and *R. ferrumequinum* have some functional similarities. However, *R. ferrumequinum* gut microbiota resulted in a lower level of activated natural killer cell ratios. This may be due to differences in the structure and function of the gut microbiota due to different genetic backgrounds, despite similar diets. Diet significantly influences the type and function of gut microbiota, and different diets induce gut microbiota to produce different metabolites and exert different immunomodulatory effects. *Hipposideros armiger* is a member of the genus *Hipposideros* in the family *Hipposiderinae*, and *R. ferrumequinum* is a member of the genus *Rhinolophus* in the family *Rhinolophinae*. These two bats, as representatives of insectivorous bats, are distantly related despite their similar dietary habits, but their gut microbiota still share similar immunomodulatory functions; therefore, it is prudent to hypothesize that the gut microbiota of insectivorous bats may all share similar functions. Whether the gut microbiota of bats with different diets has similar functions needs to be confirmed by further studies.

## 5. Conclusions

In conclusion, this study demonstrates that the gut microbiota of *R. ferrumequinum* may modulate the host immune system by altering immune cells involved in both innate and adaptive immunity. In addition, our results suggest some similarities in the immunomodulatory functions of the gut microbiota in insectivorous bats. However, a shortcoming of this study is that the function of the bat gut microbiota was obtained from a mouse model and was not validated in the bats themselves, which means that the results obtained do not directly prove the same function in bats. We emphasize that bat gut microbiota may represent a new probiotic reservoir, suggesting that it is feasible to screen for probiotics with immunomodulatory functions from bat gut microbiota. The results of this study deepen our knowledge of bat immunization, which will contribute to the prevention and control of emerging infectious diseases and the protection of human and animal health. This study also serves as a reference for the study of other wildlife gut microbiota. In the future, further extended and in-depth explorations targeting the structure and function of bat gut microbiota are needed.

## Data Availability

The data for this study are available from the NCBI Sequence Read Archive for the biological project PRJNA1210788.

## Supplementary Materials

The following supporting information can be downloaded at https://www.mdpi.com/article/10.3390/ani15050685/s1: Figure S1: Changes in the gut microbiota profiles of mice after FMT; Figure S2: Gate of flow cytometry.
