# Supplementary material for: The Gut Microbiota of the Greater Horseshoe Bat Confers Rapidly Corresponding Immune Cells in Mice
Source: Animals (Basel). 2025 Feb 26;15(5):685. doi: 10.3390/ani15050685 (PMC11899282; doi:10.3390/ani15050685)
Supplement: Supplementary file 1 [file animals-15-00685-s001.zip › animals-3466561-supplementary.pdf]

Supplementary Material

Supplementary Figures

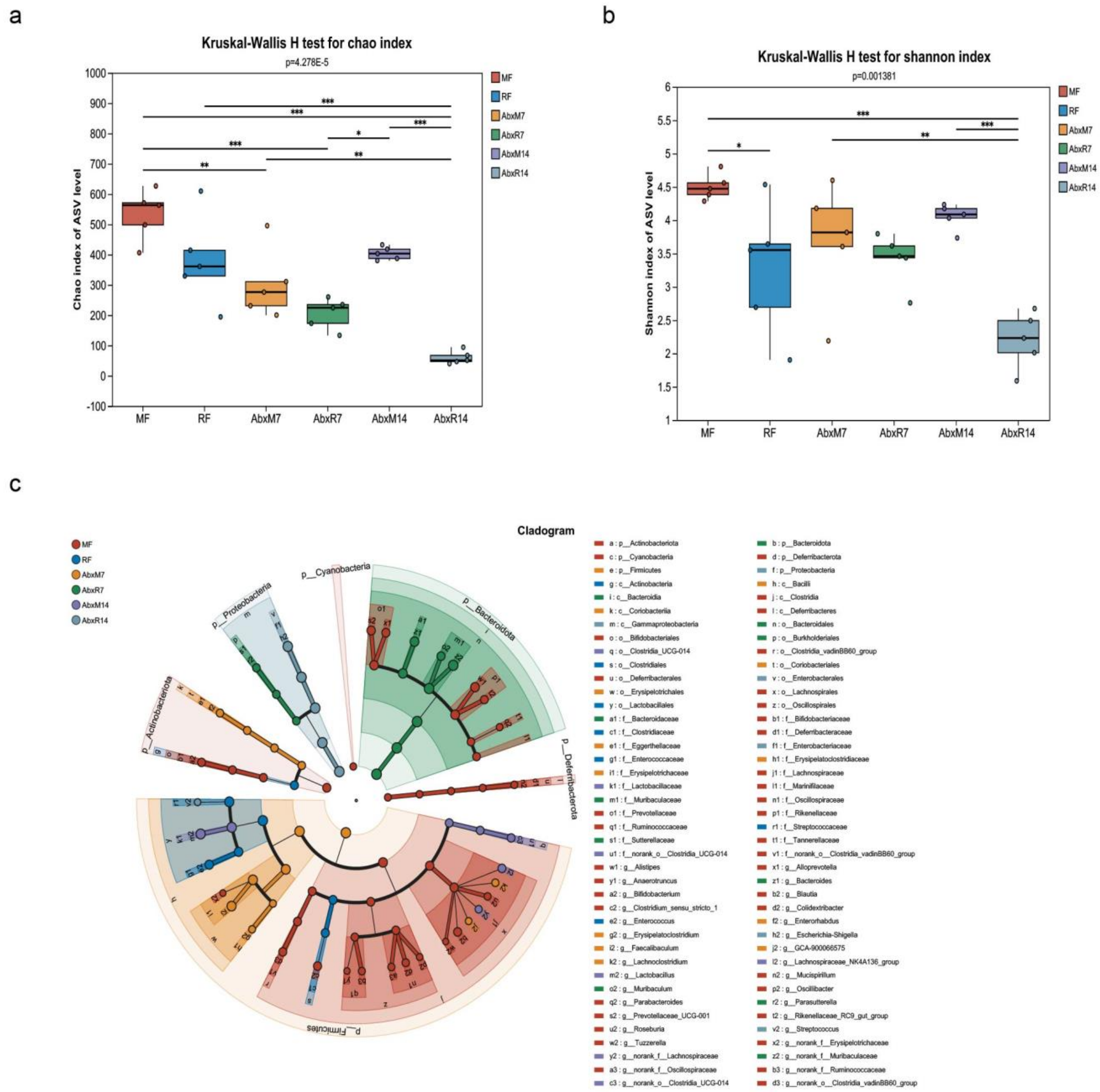

Figure S1. Changes in the gut microbiota profiles of mice after FMT.

Results in chao index(a) and Shannon index(b). Linear discriminant analysis effect size (LEfSe) circular cladogram between the different groups(c).

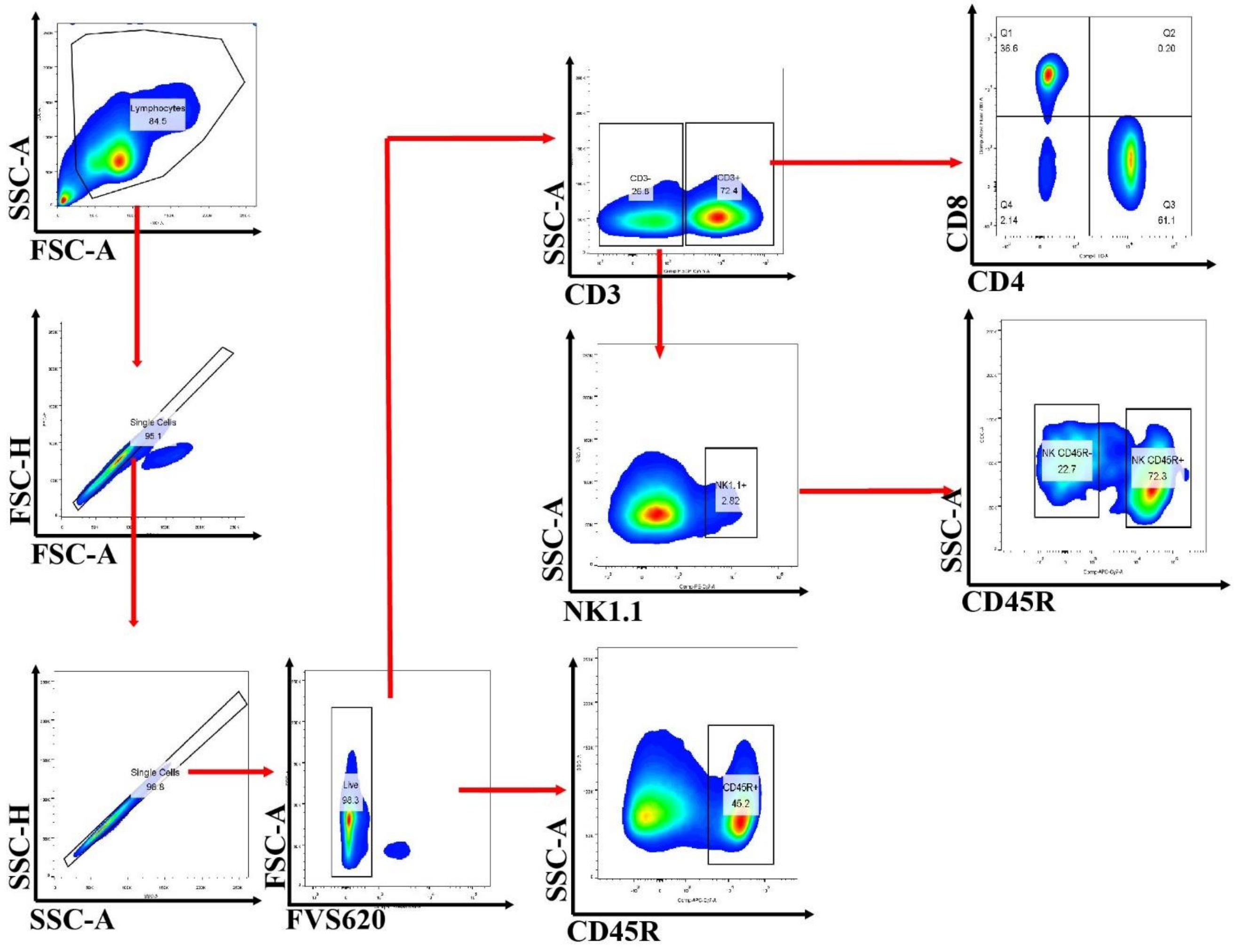

Figure S2. Gate of flow cytometry.
